# Cough syncope: A familiar stranger

**DOI:** 10.1002/kjm2.12830

**Published:** 2024-05-18

**Authors:** Nan Wang, Qing Zhang, Jia‐Fu Wei

**Affiliations:** ^1^ Department of Cardiology West China Hospital, Sichuan University Chengdu China

A 65‐year‐old man presented with recurrent syncope, with episodes occurring almost daily over the previous month. These episodes were typically preceded by a dry cough. Moreover, seizure‐like manifestations accompanied the episodes, as reported by witnesses. He had a 15‐, 8‐, and 2‐year history of hypertension, diabetes, and chronic pulmonary disease (COPD), respectively; for these conditions, he was routinely taking benazepril, metformin, and nifedipine, respectively.

On admission, physical examination and routine blood testing revealed normal findings. No significant structural cardiac disease or arrhythmia was noted in the Holter monitoring and echocardiography findings. Chest computed tomography (CT) revealed signs of COPD (Figure 1A). CT angiography demonstrated significant stenosis in the triple coronary arteries and mild stenosis in the bilateral internal carotid arteries (Figure 1B–D). Nevertheless, brain magnetic resonance imaging revealed no abnormalities. During hospitalization, the patient experienced several instances of typical, rapid cough syncope onset: bouts of coughing, facial congestion, and limb jerking, followed by recovery of normal consciousness immediately after coughing cessation. During cough syncope onset, the patient did not demonstrate a significant decrease in blood pressure (BP) or oxygen saturation; however, a slight increase in heart rate (HR) was recorded. Furthermore, electroencephalography revealed no epileptic activity even during syncope onset (Figure 1E).

**FIGURE 1:**
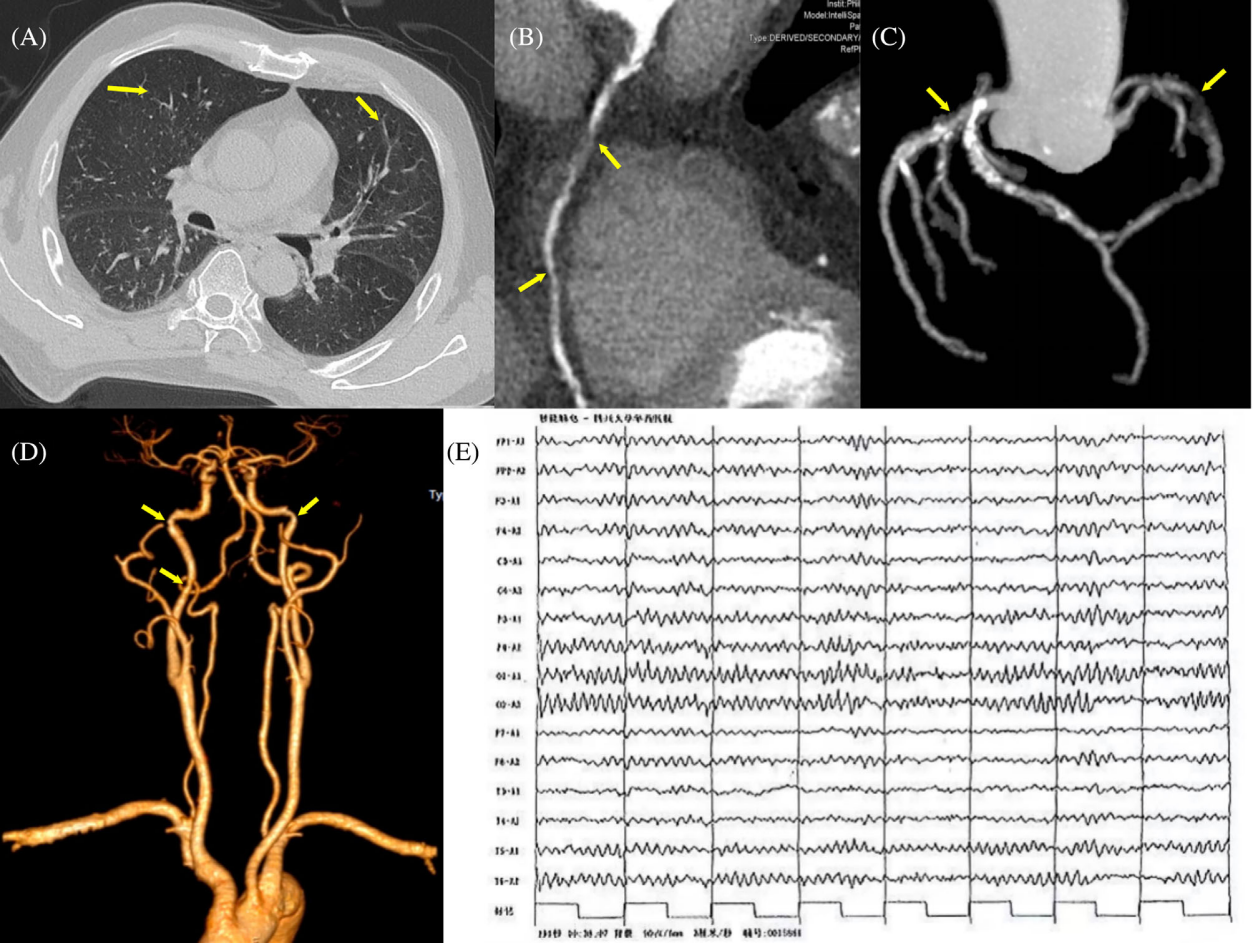
(A) Chest CT showing a “barrel chest” and emphysema (the yellow arrows). (B), (C) Coronary CT angiography showing significant calcification in the left coronary artery, including the anterior descending artery and the circumflex branch (B), as well as 80% stenosis in the right coronary artery (C) (the yellow arrows). (D) Neck CT angiography showing mild stenosis of the bilateral internal carotid arteries (the yellow arrows). (E) Electroencephalogram taken during a patient‐reported cough syncope episode showing normal results. CT, computer tomography.

Percutaneous coronary intervention was performed, with stents implanted in the involved arteries. Statin and antiplatelet treatments were also initiated. In addition, an antitussive drug was prescribed to alleviate the cough. Because dry cough is a side effect of benazepril, the patient's routine benazepril prescription was replaced with that for amlodipine. The cough syncope frequency decreased gradually, and coughing ceased after 2 weeks of treatment.

Cough syncope, accounting for 6% of all hospital admissions,^1^ is not difficult to diagnose because of its typical onset features. However, its pathophysiological mechanisms remain unclear. Initially, the condition was called “laryngeal vertigo” or “laryngeal epilepsy” because it can manifest a paroxysm of coughing, followed by fainting or vertigo with or without convulsions. However, one previous study revealed that only one of five cough syncope cases was noted to show abnormal electroencephalography findings suggestive of epilepsy.^2^ Hemodynamic theory suggests that the high intrathoracic pressure generated during coughing leads to a critical decrease in venous return and cardiac output; it also stimulates arterial baroreceptors, resulting in peripheral vasodilation and further reduction in BP.^3^ The decrease in cough‐induced systolic BP tends to be greater in patients with cough syncope than in those with vasovagal syncope. Moreover, baroreflex activation may induce bradycardia arrhythmia. For instance, Waldmann et al. reported that a healthy 42‐year‐old man experienced paroxysmal complete atrioventricular block during coughing. In contrast, our patient did not exhibit a considerable decrease in BP or HR; therefore, other mechanisms, such as an increase in intrathoracic pressure leading to a reduction in cerebral perfusion, may underlie cough syncope development.^4^

Addressing relevant etiologies and triggers is essential for cough management. In addition to COPD, the use of benazepril—an angiotensin‐converting enzyme inhibitor (ACEI)—may have led to cough syncope development in our patient. Although our patient appeared to tolerate benazepril well over several years, he may have experienced late‐onset side effects of benazepril. For instance, in 45 patients with ACEI angioedema, the duration of ACEI therapy before angioedema onset was noted to vary from 1 day to 5 years.^5^

Despite cough syncope being a well‐known condition, consensus regarding appropriate methods for its evaluation and management is lacking. Hemodynamic monitoring and electroencephalography during relevant episodes may assist in diagnosing cough syncope and understanding the underlying mechanisms; cough management may also be prioritized.

## CONFLICT OF INTEREST STATEMENT

All authors declare no conflict of interest.

## References

[1] Grossman SA , Fischer C , Lipsitz LA , Mottley L , Sands K , Thompson S , et al. Predicting adverse outcomes in syncope. J Emerg Med. 2007;33(3):233–239. 17976548 10.1016/j.jemermed.2007.04.001 PMC2276584

[2] O'Doherty DS . Tussive syncope and its relation to epilepsy. Neurology. 1953;3(1):16–21. 13013494 10.1212/wnl.3.1.16

[3] Dickinson O , Akdemir B , Puppala VK , Krishnan B , Detloff BLS , Sakaguchi S , et al. Blunted chronotropic response to hypotension in cough syncope. JACC Clin Electrophysiol. 2016;2(7):818–824. 29759766 10.1016/j.jacep.2016.02.017

[4] Linzer M , McFarland TA , Belkin M , Caplan L . Critical carotid and vertebral arterial occlusive disease and cough syncope. Stroke. 1992;23(7):1017–1020. 1615535 10.1161/01.str.23.7.1017

[5] Sondhi D , Lippmann M , Murali G . Airway compromise due to angiotensin‐converting enzyme inhibitor‐induced angioedema: clinical experience at a large community teaching hospital. Chest. 2004;126(2):400–404. 15302724 10.1378/chest.126.2.400

